# Supplementary material for: Autoregulation of yeast ribosomal proteins discovered by efficient search for feedback regulation
Source: Commun Biol. 2020 Dec 11;3:761. doi: 10.1038/s42003-020-01494-z (PMC7732827; doi:10.1038/s42003-020-01494-z)
Supplement: Supplementary file 1 — Supplementary Information [file 42003_2020_1494_MOESM1_ESM.pdf]

Supplementary Information for

Autoregulation of Yeast Ribosomal Proteins Discovered by Efficient Search for Feedback Regulation

Basab Roy[1], David Granas[1], Fredrick Bragg, Jonathan Cher, Michael White, Gary D Stormo

Department of Genetics and Center for Genome Sciences and Systems Biology

Washington University School of Medicine, St Louis, MO 63110 USA

[1] these authors contributed equally

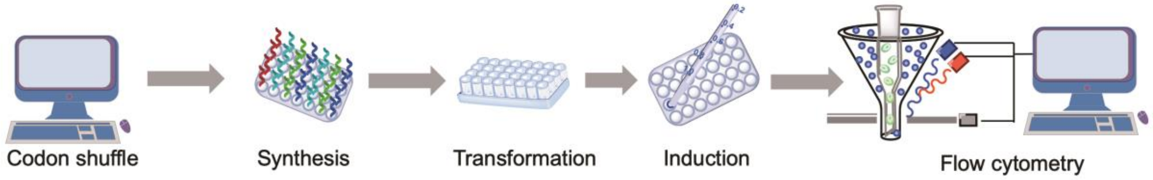

Supplementary Figure S1: Scheme for testing autoregulation. The cre-less genes were synthesized without cis regulatory elements, such as introns and UTRs, and their codons were shuffled to eliminate the chance of autoregulation by the cre-less protein. The cre-less genes were cloned into the MJB1 vector driven by the GAL1 promoter. Each cre-less gene plasmid was transformed into a yeast strain that had a GFP-tagged version of the corresponding gene at its native locus. Transformations were done either individually or in 96-well plate format. The cre-less protein was induced by adding galactose. The level of cre-less protein and the native protein were monitored by mCherry and GFP fluorescence, respectively. (This figure created using ChemDraw from PerkinElmer Informatics.)

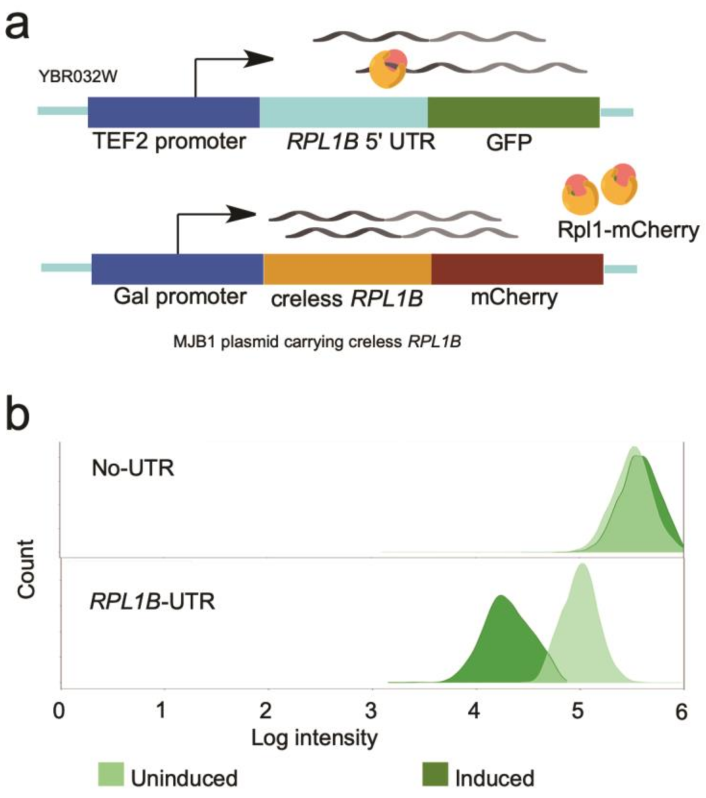

Supplementary Figure S2: Reporter gene assay with *RPL1B* UTR. (**a**) A reporter construct was made by placing the *RPL1B* 5' UTR upstream of GFP and having it driven by the *TEF2* promoter. A negative control construct was also made without the *RPL1B* 5' UTR. Each construct was integrated in the genome (Chromosome II, YBR032W) and the cells were transformed with the cre-less *RPL1B* plasmid. (**b**) The cre-less *RPL1B* was induced with galactose and GFP fluorescence was measured to detect autoregulation. A decrease in GFP after induction only occurred with the construct containing the *RPL1B* 5' UTR showing it is sufficient to allow autoregulation by Rpl1.

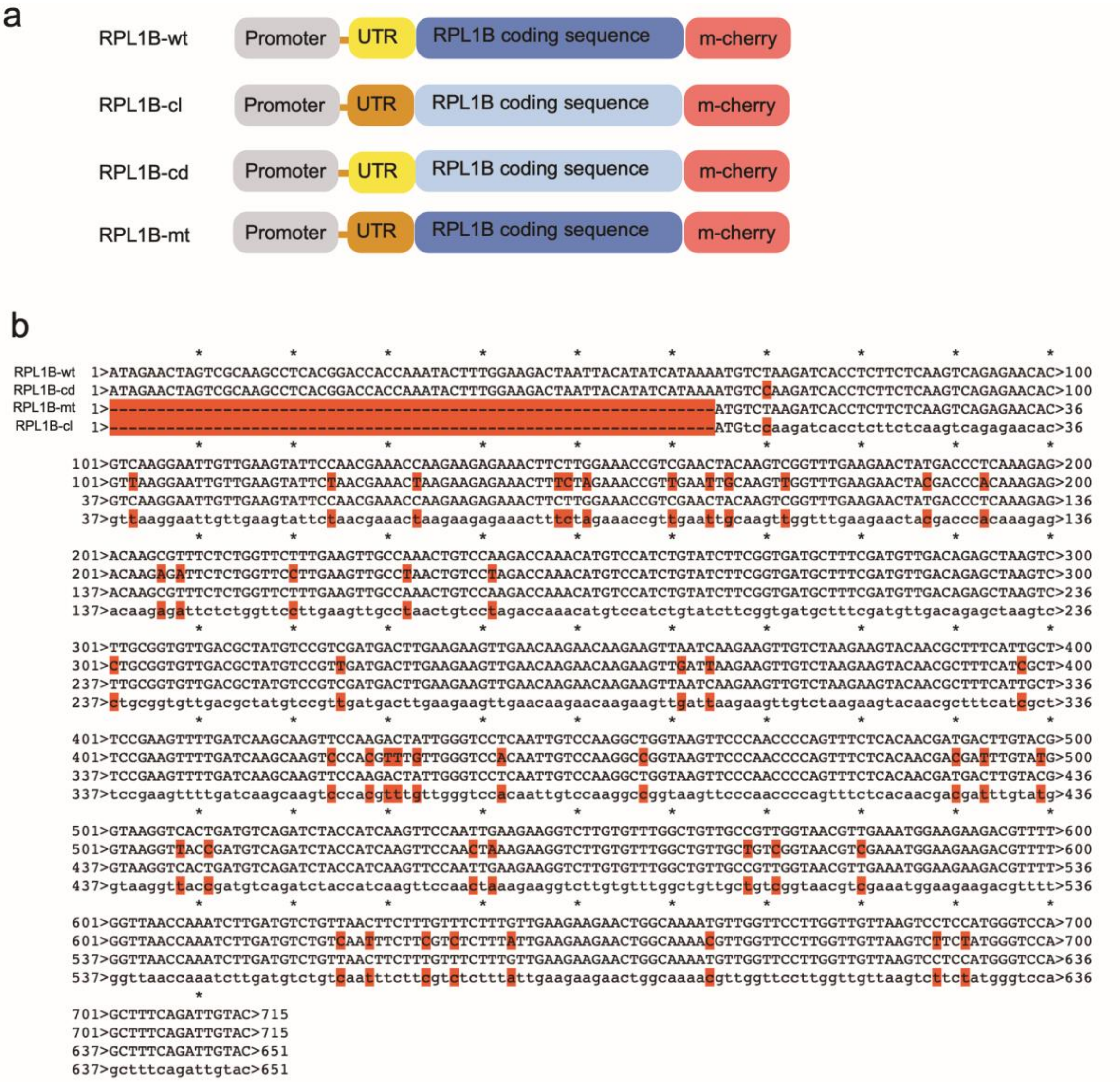

Supplementary Figure S3: Design and sequence alignment of *RPL1B* variants used to compare the contribution of regulatory elements in protein autoregulation. Beside the cre-less version (RPL1B-cl) three other alternative structures were synthesized: wild-type for both the 5'UTR and coding region (RPL1B-wt); wild-type for the 5'UTR with a shuffled coding region (RPL1B-cd); and mutant 5'UTR but wild-type coding region (RPL1B-mt). All of the three variants were tagged with mCherry and cloned in the MJB1 vector for expression by galactose induction. (**a**) Cartoon of the *RPL1B* gene constructs with different elements, where differences in sequences within each region are indicated by altered colors. (**b**) Sequence alignments of the four *RPL1B* constructs with mismatches highlighted in red.

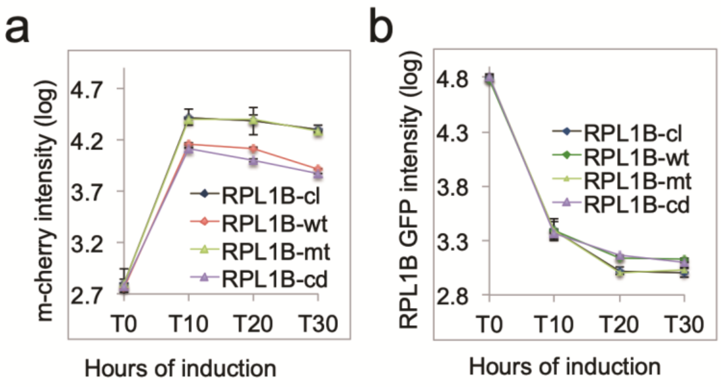

Supplementary Figure S4: The mCherry and GFP expression levels of RPL1B-cl, RPL1B-wt, RPL1B-cd and RPL1B-mt after 10, 20 and 30 hours of galactose induction. (**a**) The mCherry signal is indicative of RPL1B-cl, RPL1B-wt, RPL1B-cd and RPL1B-mt from the MJB1 plasmid. (**b**) The GFP signal indicates the expression level of the chromosomal copy of *RPL1B*

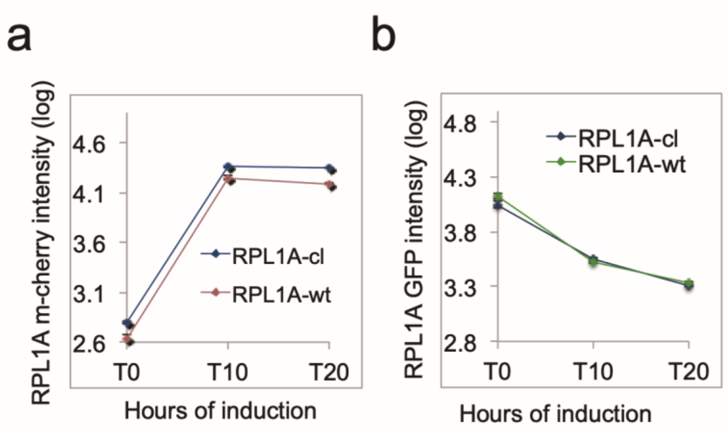

Supplementary Figure S5: The mCherry and GFP expression levels of RPL1A-cl and RPL1A-wt after 10 and 20 hours of galactose induction. (**a**) The mCherry signal is indicative of RPL1A-cl and RPL1A-wt from the MJB1 plasmid. (**b**) The GFP signal indicates the expression level of the chromosomal copy of *RPL1A*.

```
RPL1B-UTR   ATAGAACTAGTCGCAAGCCTCACGGACCACCAAATACTTTGGAAGACTAATTACATATCATAAAATGTCT

Control-UTR AGTAACAAAAAATTAAAGTTAATTAAGGAGATTAAACTATATATCAACAAAAAATTGTTAATATACCTCT
```

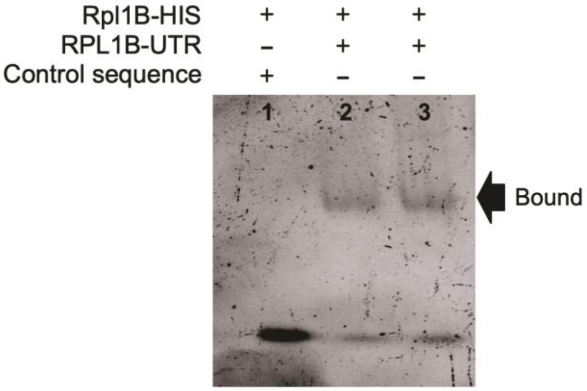

Supplementary Figure S6: EMSA with purified his-tagged Rpl1 (Rpl1B-HIS) and either the control sequence (lane 1), synthetic *RPL1B*-UTR RNA (lane 2) or *in-vitro* transcribed *RPL1B*-UTR (lane 3).

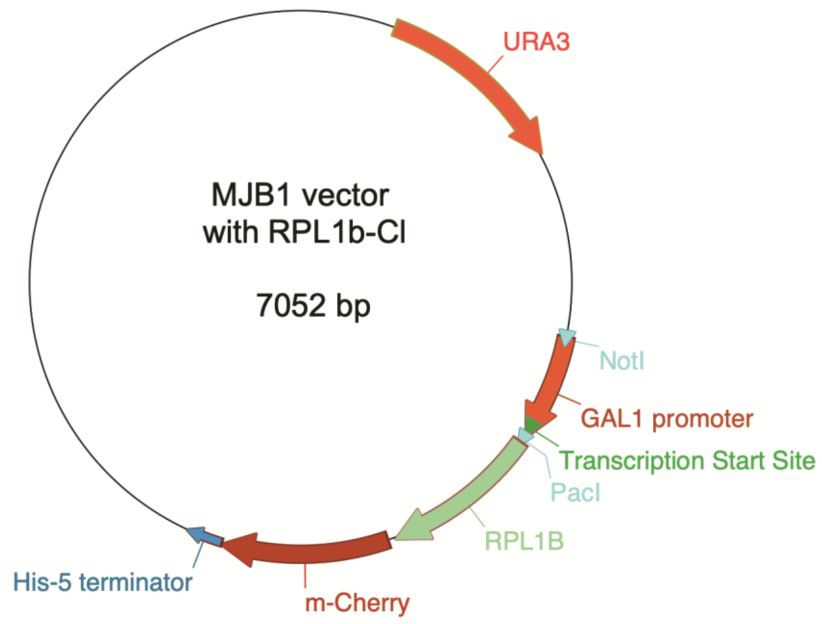

Supplementary Figure S7: The MJB1 vector with the expression cassette including GAL1 promoter, mCherry fused cre-less *RPL1B* (RPL1B-cl) and His-5 terminator.

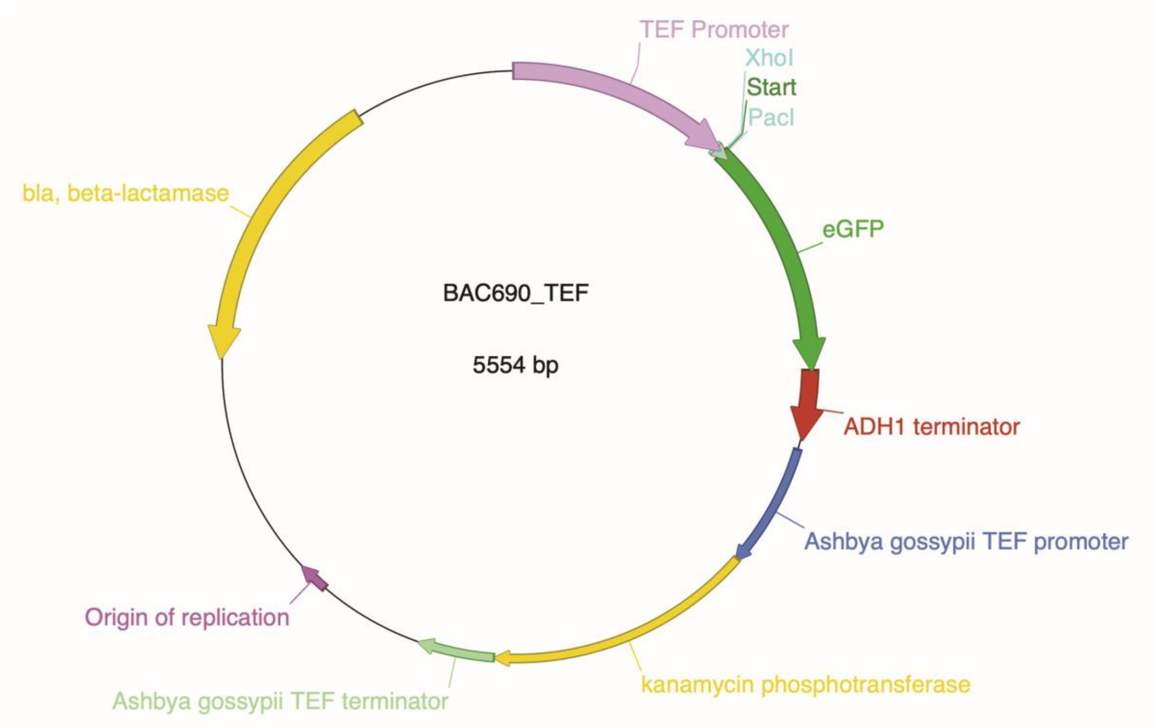

Supplementary Figure S8: The BAC690_TEF vector map showing the TEF2 promoter, cloning location (between XhoI and PacI sites) for placing UTRs from either *RPS22B* or *RPL1B* upstream of GFP and the ADH1 terminator. The whole cassette and the kanamycin phosphotransferase was amplified with flanking regions homologous to the site of insertion in the yeast genome (YBRO32W).
