# Supplementary material for: Autoregulation of yeast ribosomal proteins discovered by efficient search for feedback regulation
Source: Commun Biol. 2020 Dec 11;3:761. doi: 10.1038/s42003-020-01494-z (PMC7732827; doi:10.1038/s42003-020-01494-z)
Supplement: Supplementary file 2 — Description of additional supplementary files [file 42003_2020_1494_MOESM2_ESM.docx]

Description of Additional Supplementary Files

Supplementary Data 1: GFP expression for each replicate of each ribosomal protein-GFP strain containing the cre-less gene plasmid under both inducing and non-inducing conditions. Data are plotted in Figure 2.

Supplementary Data 2: GFP expression of each replicate of each ribosomal protein-GFP strain without the cre-less plasmid under both inducing and non-inducing conditions.

Supplementary Data 3: GFP expression of each replicate of the variants of the RPS22b 5'UTR driving GFP, under inducing and non-inducing conditions. Data are plotted in Figure 3.

Supplementary Data 4: GFP expression of each replicate for variants of the RPL1b 5' UTR under inducing and non-inducing conditions. Data are plotted in Figure 4.

Supplementary Data 5: Sequences of Codon-shuffled yeast genes.
